# Airway Delivery of Anti-influenza Monoclonal Antibodies Results in Enhanced Antiviral Activities and Enables Broad-Coverage Combination Therapies

**DOI:** 10.1128/JVI.00052-20

**Published:** 2020-10-27

**Authors:** Adam Vigil, Natalia Frias-Staheli, Teresa Carabeo, Michael Wittekind

**Affiliations:** a ContraFect Corporation, Yonkers, New York, USA; University of North Carolina at Chapel Hill

**Keywords:** antibody, combination therapy, influenza, neutralizing antibodies

## Abstract

Effective and reliable anti-influenza treatments are acutely needed and passive immunizations using broadly neutralizing anti-influenza monoclonal antibodies (bNAbs) are a promising emerging approach. Because influenza infections are initiated in and localized to the pulmonary tract, and newly formed viral particles egress from the apical side of the lung epithelium, we compared the effectiveness of hemagglutinin (HA) stalk-binding bNAbs administered through the airway (intranasal or via nebulization) versus the systemic route (intraperitoneal or intravenous). Airway deliveries of various bNAbs were 10- to 50-fold more effective than systemic deliveries of the same bNAbs in treating H1N1, H3N2, B/Victoria-, and B/Yamagata-lineage influenza viral infections in mouse models. The potency of airway-delivered anti-HA bNAbs was highly dependent on antiviral neutralization activity, with little dependence on the effector function of the antibody. In contrast, the effectiveness of systemically delivered anti-HA bNAbs was not dependent on antiviral neutralization, but critically dependent on antibody effector functions. Concurrent administration of a neutralizing/effector function-positive bNAb via the airway and systemic routes showed increased effectiveness. The small amount of airway-delivered bNAbs needed for effective influenza treatment creates the opportunity to combine potent bNAbs with different anti-influenza specificities to generate a cost-effective antiviral therapy that provides broad coverage against all circulating influenza strains infecting humans. A 3 mg/kg dose of the novel triple antibody combination CF-404 (i.e., 1 mg/kg of each component bNAb) delivered to the airway was shown to effectively prevent weight loss and death in mice challenged with ten 50% lethal dose (LD_50_) inoculums of either H1N1, H3N2, B/Victoria-lineage, or B/Yamagata-lineage influenza viruses.

**IMPORTANCE** Influenza causes widespread illness in humans and can result in morbidity and death, especially in the very young and elderly populations. Because influenza vaccination can be poorly effective some years, and the immune systems of the most susceptible populations are often compromised, passive immunization treatments using broadly neutralizing antibodies is a promising therapeutic approach. However, large amounts of a single antibody are required for effectiveness when delivered through systemic administration (typically intravenous infusion), precluding the feasible dosing of antibody combinations via this route. The significance of our research is the demonstration that effective therapeutic treatments of multiple relevant influenza types (H1N1, H3N2, and B) can be achieved by airway administration of a single combination of relatively small amounts of three anti-influenza antibodies. This advance exploits the discovery that airway delivery is a more potent way of administering anti-influenza antibodies compared to systemic delivery, making this a feasible and cost-effective therapeutic approach.

## Abstract (precis)

Influenza causes widespread illness in humans and can result in morbidity and death, especially in the very young and elderly populations. Because influenza vaccination can be poorly effective some years, and the immune systems of the most susceptible populations are often compromised, passive immunization treatments using broadly neutralizing antibodies is a promising therapeutic approach. However, large amounts of a single antibody are required for effectiveness when delivered through systemic administration (typically intravenous infusion), precluding the feasible dosing of antibody combinations via this route. The significance of our research is the demonstration that effective therapeutic treatments of multiple relevant influenza types (H1N1, H3N2, and B) can be achieved by airway administration of a single combination of relatively small amounts of three anti-influenza antibodies. This advance exploits the discovery that airway delivery is a more potent way of administering anti-influenza antibodies compared to systemic delivery, making this a feasible and cost-effective therapeutic approach.

## INTRODUCTION

Influenza infections continue to cause significant illness and death every year (1), with recent influenza-associated respiratory death estimates ranging between approximately 291,000 to 646,000 per year worldwide (2). Additionally, the potential for devastating influenza pandemics across the human population is high (3). The limited effectiveness of influenza vaccines is primarily due to the ability of the virus to rapidly mutate, giving rise to new variations each year that necessitate annual reformulations of the vaccine (4). Currently available treatment options, such as neuraminidase inhibitors, suffer from resistance (5) and the limited postinfection time frame in which they are effective (6). There remains an acute need for new anti-influenza therapeutics that provide a high degree of effectiveness and broad coverage of the influenza strains that infect humans.

Antibodies to influenza viruses can be effective therapeutically. Passive immunization using hyperimmune serum derived from convalescent plasma of patients who recovered from infections caused by the pandemic 2009 H1N1 influenza virus has been shown to be effective in suppressing viral load, as well as improving survival in infected patients (7). Monoclonal antibodies (MAbs) that recognize the head region of the influenza viral hemagglutinin (HA) protein have been shown to be effective in treating influenza infections in animal models (8, 9). However, HA head-specific MAbs are generally not broadly reactive across many influenza virus isolates (with notable exceptions [10, 11]), due to the large degree of strain-to-strain variability in the amino acid residues within the HA head region (12). An effective alternative approach is to identify MAbs that bind to the highly conserved stalk region of the influenza HA protein (13). These stalk-specific MAbs are often broadly neutralizing antibodies (bNAbs), as they recognize the highly conserved epitopes within the HA stalk region. They prevent viral infection by inhibiting the pH-sensitive conformational change of the HA protein essential for the virus/cell fusion event (14), and require Fc gamma receptor engagement for full activity when administered systemically (9, 15).

Perhaps because of the effectiveness of the classic systemic passive immunization approaches using infused antisera, the general approach to delivering anti-influenza MAbs has been via the systemic route. However, because infections caused by influenza viruses are initiated in, and generally localized to, the respiratory tract (16), it is reasonable to consider the administration of anti-influenza virus therapies directly to the patient’s airway. In an early study, Akerfeldt et al. observed approximately 50-fold enhanced efficacy of airway delivery over systemic delivery when using human gamma globulin to treat influenza infections in mice (17). Clinically, airway delivery for influenza therapy has been employed for the administration of the marketed neuraminidase inhibitor zanamivir (18), and in clinical trials for DAS-181, an enzyme-based sialidase fusion protein (19). Airway delivery of purified anti-influenza MAbs has not been clinically tested to date.

Administration of MAbs (or antibody-like derivatives) directly to the airway through inhalation for treating respiratory diseases has been characterized for a number of systems (see reference 20 for review and the references within). Generally, the results of these studies indicate that exposure to aerosolized MAbs is well tolerated, with no or minor impact on activity of the therapeutic (21). For example, airway delivery of cetuximab has been proposed for treating lung cancer (22) and studies indicate that this antibody can be nebulized successfully with little effect on activity (23). Airway-delivered nebulized anti-VEGF murine IgG2a MAb G6-31 has been shown to be effective in models of primary pulmonary adenocarcinoma (24). An anti-IL-13 antibody Fab fragment can be nebulized with no loss of activity (25) and airway delivery of the Fab has been shown to be effective in models of chronic asthma (26). ALX-0171, an inhaled anti-RSV camelidae-derived nanobody, has been tested in clinical trials and appears to be well tolerated in humans (27). For anti-influenza treatment, HA stalk-binding bNAbs delivered intranasally have been shown to be effective in murine models of influenza (28, 29).

In this study, we evaluated the attributes necessary for anti-influenza viral effectiveness for both airway- and systemic–delivered bNAbs. The findings indicate that the neutralization and effector function engagement requirements are different for anti-influenza antibodies delivered via the airway versus those administered by the systemic route. Because of the orthogonal mechanisms of action of the two administration routes, we found relatively small amounts of effector function-positive bNAbs to be highly effective when dosed via the airway and systemic routes simultaneously. Given that the potency of airway-delivered bNAbs is at least one log greater than that of systemic-delivered bNAbs, there is an opportunity to combine several bNAbs and deliver them at relatively low doses via the airway to achieve broad coverage against multiple influenza strains. Here, we characterize CF-404, an inhaled three-bNAb combination that effectively treats infections caused by the circulating influenza strains commonly infecting humans, covering influenza A (IAV) groups 1 and 2 and both lineages of influenza B (IBV).

## RESULTS

### Airway delivery of anti-influenza bNAbs is more effective than systemic delivery for treating IAV and IBV infections.

**FIG 1:**
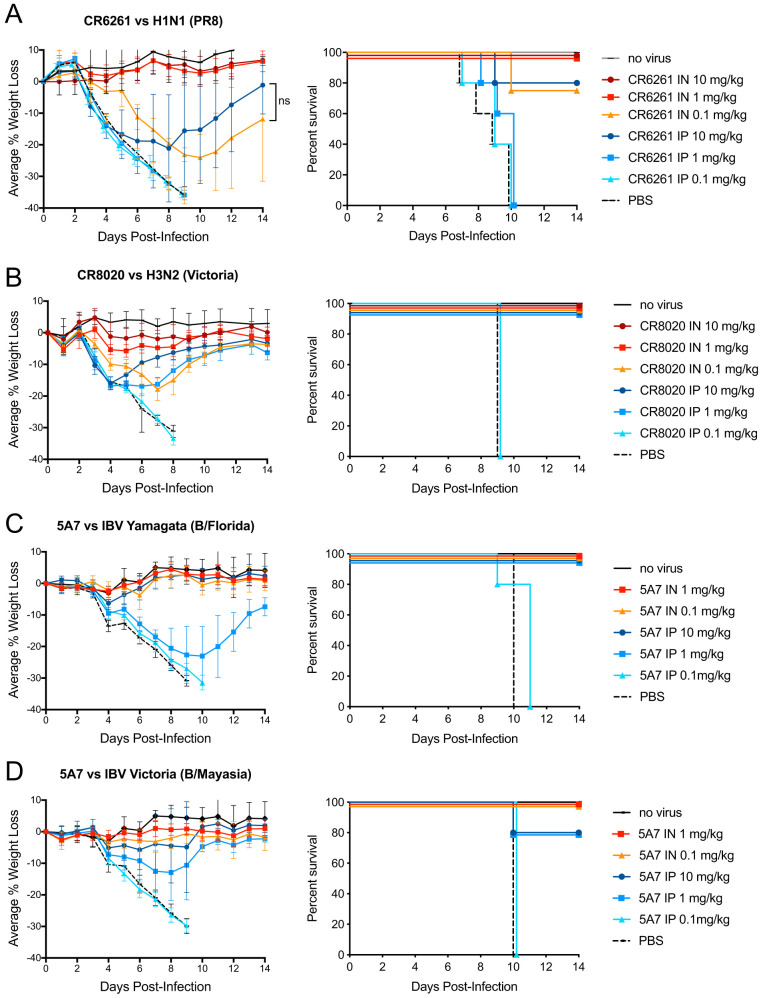
Airway delivery of anti-influenza bNAbs is more effective than systemic delivery for treating IAV and IBV infections. All dosing cohorts (*n* = 5) were subjected to uniform 10× LD_50_ viral challenges with various influenza strains. bNAbs were tested at various dosing levels by either the i.n. or i.p. routes of administration at 24 h postinfection. Each panel shows the percent changes in body weight over 14 days with error bars set at 1 standard deviation (SD) (left) and the Kaplan-Meier survival plots (right). (A) bNAb CR6261* against H1N1 influenza strain A/Puerto Rico/8/34 (PR8); (B) bNAb CR8020* against H3N2 influenza strain A/Victoria/361/2011; (C) bNAb 5A7* against IBV strain B/Florida/04/2006 (Yamagata lineage); (D) bNAb 5A7* against IBV strain B/Malaysia/2506/2004 (Victoria lineage). ns, not significant.

Anti-influenza antibodies CR6261 (30), CR8020 (31), and 5A7 (32) were generated containing protein sequences identical to published sequences and are designated CR6261*, CR8020*, and 5A7*, respectively (see the Materials and Methods section). These antibodies are human IgG1 and should exhibit similar relative FcγR-mediated biological activities in mouse models of disease (33). Using a mouse model of influenza infection, we compared the therapeutic effectiveness of the anti-IAV-group 1 stalk-binding bNAb CR6261* (30) when delivered by different routes of administration. With CR6261* administered at 24 h post-H1N1 viral infection, improvements in molar potency on the order of greater than 50-fold were observed for intranasal (i.n.) delivery compared to systemic delivery via the intraperitoneal (i.p.) route (Fig. 1 A). The weight loss profiles of the 10 mg/kg i.p.- and 0.1 mg/kg i.n.- dosed cohorts, as assessed by area under the concentration-time curve (AUC) analysis, were not significantly different, although the shape of the curves are clearly distinct (see the Discussion section). When a similar experiment was performed using a potent IAV-group 2 H3N2 virus and treatment with the stalk-binding anti-IAV-group 2 bNAb CR8020* (31), improvements in potency for the i.n.-delivered bNAb over the same bNAb delivered via the i.p. route was greater than 10-fold (Fig. 1 B). Similarly, when the anti-IBV stalk-binding bNAb 5A7* (32) was used to treat either IBV-Victoria (Fig. 1 C) or Yamagata (Fig. 1 D) lineage infections, improvements in potency for the i.n.-delivered bNAb over the same bNAb delivered via the i.p. route was greater than 50-fold.

These results demonstrate that anti-influenza stalk-binding bNAbs delivered via the i.n. route are more effective than the same amount of antibody delivered via the i.p. route, and that this finding holds true for treatment of infections due to both groups 1 and 2 IAVs, as well as for viruses of both major IBV lineages.

**FIG 2:**
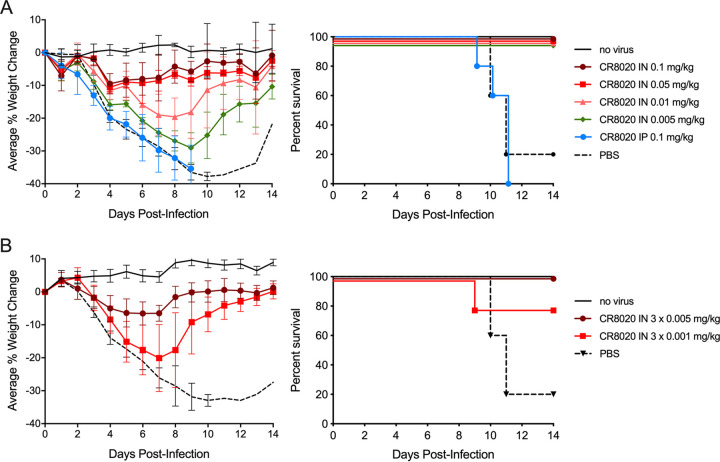
Dose responses for treating IAV H3N2 infection with either single or multiple doses of bNAb via the airway. All dosing cohorts (*n* = 5) were subjected to a uniform 10× LD_50_ H3N2 viral challenge (strain A/Victoria/361/2011). bNAb CR8020* was tested at various dosing levels by the i.n. route of administration at 8 h postinfection. Each panel shows the percent changes in body weight over 14 days with error bars set at 1 SD (left) and the Kaplan-Meier survival plot (right). (A) Mice cohorts received single i.n. doses of 0.10, 0.05, 0.01, or 0.005 mg/kg. Control groups were uninfected, PBS-treated, and single dose of 0.1 mg/kg i.p. (B) Mice cohorts received three equal i.n. doses of 0.005 mg/kg (0.015 mg/kg total) or 0.001 mg/kg (0.003 mg/kg total) at 8, 32, and 56 h postinfection.

Using the H3N2 viral infection model with bNAb administration at 8 h postinfection, we investigated the dose-response relationship of CR8020* bNAb i.n. treatments. As shown in Fig. 2A, under these conditions single doses of as little as 0.005 mg/kg i.n. showed protection from weight loss and lethality, while doses of 0.10 mg/kg i.p. did not. In the same model, when the bNAb was administered as three daily low-level i.n. doses, 5% and 20% maximal weight losses were observed for the 0.015 and 0.003 mg/kg total doses, respectively (Fig. 2 B), and, although 4 of 5 mice survived in the 0.003 mg/kg total dose group, the difference between survival of that group and the phosphate-buffered saline (PBS)-dosed control group did not reach statistical significance in this experiment (*P* = 0.142).

### Anti-influenza antibody activity in the airway is dependent on viral neutralization activity.

To determine whether the ability of antibodies to directly neutralize the influenza virus is an important factor for i.n. and i.p. anti-influenza activities, two antibodies differing in their viral neutralization profiles were compared in the mouse influenza H3N2 infection model. The neutralizing antibody tested was CR8020*, a stalk-binding antibody that has been shown to effectively neutralize a broad set of H3N2 viruses (31). The nonneutralizing antibody tested was 6P15, an antibody that binds to the viral HA protein of all H3N2 strains tested but does not neutralize the viruses. Both antibodies were constructed to have human IgG1 Fc sequences.

**FIG 3:**
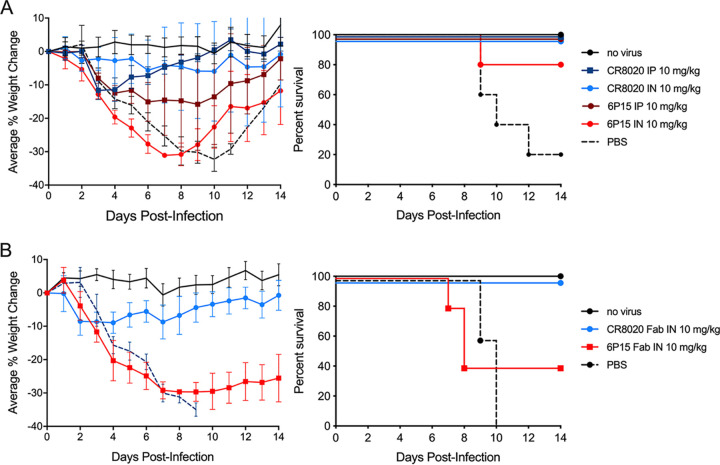
Anti-influenza antibody activity in the airway is dependent on viral neutralization activity. All dosing cohorts (*n* = 5) were subjected to a uniform 10× LD_50_ H3N2 viral challenge (strain A/Victoria/361/2011). The panels show the percent changes in body weight over 14 days with error bars set at 1 SD (left) and the Kaplan-Meier survival plot (right). Control groups were uninfected and PBS-treated infected. (A) Doses of 10 mg/kg of bNAb CR8020* and antibody 6P15 were dosed at 24 h postinfection by either the i.n. or i.p. routes of administration. (B) Mice were treated with 10 mg/kg i.n. of the Fab forms of CR8020* and 6P15.

As shown in Fig. 3A, for the systemic route of administration, the two different antibodies dosed at 10 mg/kg i.p. yielded maximal average weight loss percentages of 11% (CR8020*) and 15% (6P15). While the nonneutralizing antibody 6P15 is not as potent in preventing weight loss as the neutralizing antibody CR8020* (P = 0.0007), the finding that antibody 6P15 can be effective when delivered systemically is consistent with published studies showing the effectiveness of systemically delivered nonneutralizing antibodies against H7N9 (34). In contrast, for the airway route of administration dosed at 10 mg/kg i.n., the two antibodies had very different antiviral activity profiles, with the nonneutralizing antibody 6P15 exhibiting greatly reduced activity (>30% maximal average weight loss percentage) compared to the high level of protective activity (<6% maximal average weight loss percentage) displayed by the neutralizing antibody CR8020* (P < 0.0001). The survival profile of the 6P15 i.n.-dosed mice was not statistically significant relative to the PBS-dosed control mice (P = 0.09). While the weight loss profiles of the i.n.- and i.p.-administered neutralizing antibody CR8020* were not significantly different (P = 0.96), for the nonneutralizing antibody 6P15, the i.p.-administered dose was significantly more potent than the i.n.-delivered dose (*P < *0.0001).

The disparity of airway delivery effectiveness between the neutralizing antibody CR8020* and the nonneutralizing antibody 6P15 was also observed when tested in the Fab format (Fig. 3B). The CR8020* Fab protected mice from H3N2-induced lethality, while the 6P15 Fab-treated mice lost more weight (*P < *0.0001) and their survival rate was not statistically differentiated from the PBS-treated control mice (*P* = 0.89).

### Anti-influenza antibody activity in the airway is independent of effector function.

The Fab forms of the antibodies are missing the Fc region, which is essential for interactions with Fc gamma receptors and C1q (35), thereby rendering Fabs unable to engage effector cell function. As seen in Fig. 3B, the Fab form of neutralizing antibody CR8020* was effective in the murine influenza model when delivered i.n., suggesting that the ability to recruit effector function plays a minor role, if any, in determining an antibody’s anti-influenza activity profile in the airway.

**FIG 4:**
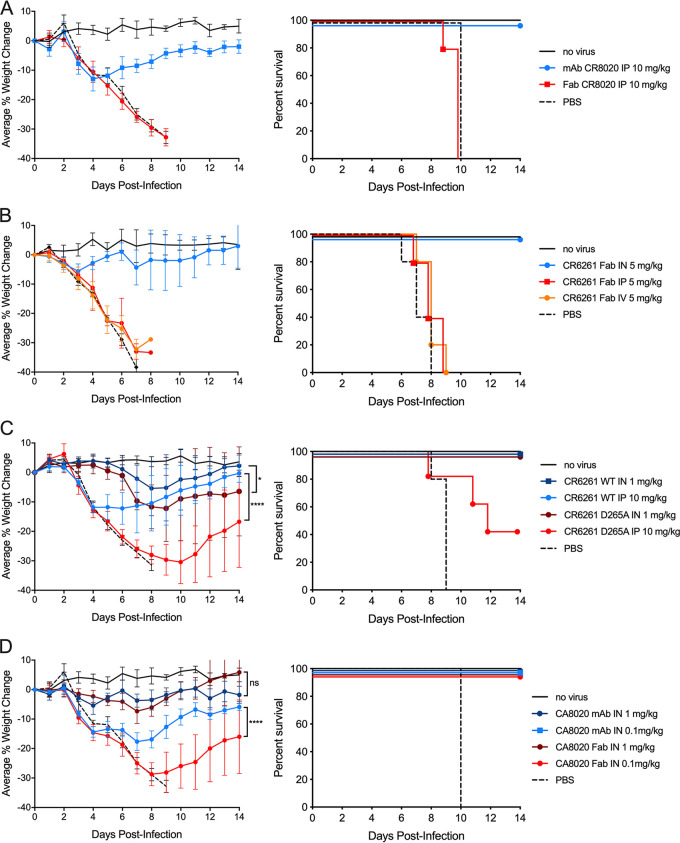
Anti-influenza antibody activity in the airway is independent of effector function. All dosing cohorts (*n* = 5) were subjected to 10× LD_50_ viral challenges and dosed at 24 h postinfection. Each experiment included two control groups: uninfected and infected with no treatment. The panels show the percent changes in body weight over 14 days with error bars set at 1 SD (left) and the Kaplan-Meier survival plot (right). (A) Mice were infected with H3N2 strain A/Victoria/361/2011 and treated with 10 mg/kg i.p. of the either the IgG1 and Fab forms of CR8020*. (B) Mice were infected with H1N1 strain A/Puerto Rico/8/34 and treated with 5 mg/kg of the Fab form of CR6261* via the i.n., i.p., and i.v routes. (C) Mice were infected with H1N1 strain A/Puerto Rico/8/34 and treated with the IgG1 form of CR6261* (with either the wild-type Fc or the D265A mutant version) via the i.n. route (dosed at 1 mg/kg) or the i.p. route (dosed at 10 mg/kg). (D) Mice were infected with H3N2 strain A/Victoria/361/2011 and treated with either 0.1 or 1.0 mg/kg i.n. of either the Fab or IgG1 forms of CR8020*. ****, *P* < 0.0001; *, *P* < 0.05; ns, not significant.

In contrast, when delivered via the i.p. route, the Fab form was completely ineffective while the full-length IgG1 form was effective (Fig. 4A), indicating that effector function is critical for potency when the antibodies are delivered systemically.

Further testing was performed in the H1N1 infection model, where the CR6261* antibody Fab fragment was shown to be effective in protecting infected mice from weight loss and death when administered via the airway, but was completely ineffective when administered via either of two different systemic routes (i.v. or i.p.) (*P < *0.0001) (Fig. 4B).

When the D265A mutation (known to disrupt the ability of the Fc to engage effector functions [36]) was introduced into the CH2 domain of the full IgG1 form of the CR6261* bNAb, the systemic activity was greatly diminished (12% versus 31% maximal average weight loss percentage [*P* < 0.0001]). Survival of the D265A mutant i.p.-treated mice was barely statistically differentiated relative to the PBS-treated mice (*P* = 0.496). The introduction of the D265 mutation affected the airway activity to a relatively smaller extent (5% versus 12% maximal average weight loss percentage [*P* = 0.035]) (Fig. 4C). The results for the i.p.-administered bNAbs are consistent with the previously demonstrated Fc gamma receptor-engagement dependency of systemically administered stalk-binding anti-HA bNAbs (9).

The IgG format appears to be more effective than the Fab format in treating H3N2 infection intranasally when tested in the dose-response experiment shown in Fig. 4D. This difference is only significant at the lowest doses tested (0.1 mg/kg) (*P < *0.0001).

### Coadministered low systemic doses enhance airway delivery effectiveness.

**FIG 5:**
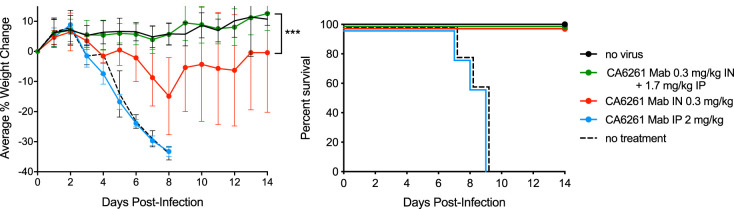
Coadministered low-level systemic doses enhance airway delivery. All dosing cohorts (*n* = 5) were subjected to a uniform 10× LD_50_ H1N1 viral challenge (strain A/Puerto Rico/8/34). Various amount of bNAb CR6261* were dosed at 24 h postinfection to three different cohorts: 2 mg/kg i.p.; 0.3 mg/kg i.n.; and 1.7 mg/kg i.p. plus 0.3 mg/kg i.n. (2 mg/kg total dose). Two control groups were included (uninfected and PBS-treated). The panels show the average percent changes in body weight over 14 days with error bars set at 1 SD (left) and the Kaplan-Meier survival plot (right). ***, *P = *0.0004.

As demonstrated in the studies shown so far, the effectiveness of airway-delivered antibodies is to a great extent neutralization-dependent and effector-function-independent, while the effectiveness of systemic-delivered antibodies is largely neutralization-independent and effector-function-dependent. These findings suggest that different mechanisms of action are driving the antiviral activities in the airway and systemic compartments. We hypothesized that administration of a neutralization-positive/effector function-positive antibody via both the i.n. and i.p. routes simultaneously might show increased overall efficacy. Using the murine H1N1 infection model and a human IgG1 form of neutralizing anti-IAV group 1 bNAb CR6121, we dosed infected mice with 2 mg/kg of CR6261* i.p. at 24 h postinfection, which was completely ineffective under these conditions (Fig. 5). In contrast, a 0.3 mg/kg i.n. dose was more effective, with a 15% maximal average weight loss percentage observed at day 8 followed by nearly complete weight recovery by day 14. Strikingly, combining the moderately effective 0.3 mg/kg i.n. dose with a completely ineffective 1.7 mg i.p. dose (total administered dose of 2 mg/kg) was highly effective, resulting in a cohort weight-loss profile indistinguishable from that of uninfected mice and significantly improved relative to the 0.3 mg/kg i.n. dose alone (*P = *0.0004). We conclude that systemic dosing, which is only effective at relatively high doses when administered alone, can confer enhanced beneficial effects when administered at lower doses in the context of concurrent treatment via the airway route.

### Airway administration protects mice when delivered prophylactically.

**FIG 6:**
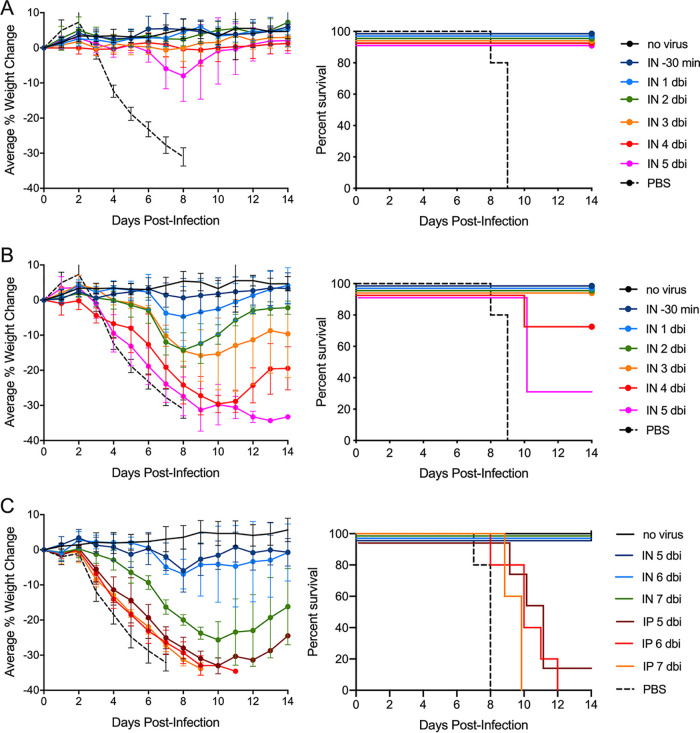
Airway administration protects mice when delivered prophylactically. Mouse cohorts (*n* = 5) were dosed with bNAb CR6261* at various times preinfection followed by a uniform 10× LD_50_ H1N1 viral challenge (strain A/Puerto Rico/8/34). In each experiment two control groups were included (uninfected and PBS-treated). (A) Single 1 mg/kg i.n. doses were administered at 5 through 1 days before infection (dbi) and at 30 min before infection. (B) Single 0.1 mg/kg i.n. doses were administered at 5 through 1 dbi and at 30 min before infection. (C) Single 1 mg/kg doses by either the i.n. or i.p. routes were administered at days 7 through 5 before infection. The panels show the percent changes in body weight over 14 days with error bars set at 1 SD (left) and the Kaplan-Meier survival plot (right).

The prophylactic activity of airway treatment was tested by i.n. administration of either 1 mg/kg or 0.1 mg/kg (panels A and B, respectively, of Fig. 6) of HA stalk-binding bNAb CR6261* to H1N1-infected mice at times ranging from 30 min to 5 days prior to infection. Complete protection from infection-induced weight loss and death was observed for the 1 mg/kg doses administered at up to 4 days preinfection, with a measurable but relatively small maximal average weight loss (<8%) becoming observable when the bNAb was delivered at 5 days preinfection. All animals survived. At the 10-fold lower dose of 0.1 mg/kg, a clear time dependency of the weight-loss profiles was observed over the 30-min to 5-day preinfection dosing time period (Fig. 6B).

When the 1 mg/kg i.n. doses were delivered at even earlier times (5, 6, and 7 days preinfection) to infected mice, administration time-dependent weight loss profiles were observed with ∼25% maximal average weight loss for the 7 day cohort, but all mice were protected from death (Fig. 6C). However, in the same experiment, mice receiving 1 mg/kg doses delivered systemically at 5, 6, and 7 days preinfection experienced maximal weight loss and all mice died except for a single mouse dosed at 5 days preinfection.

### bNAb airway delivery extends effective treatment window relative to oseltamivir.

**FIG 7:**
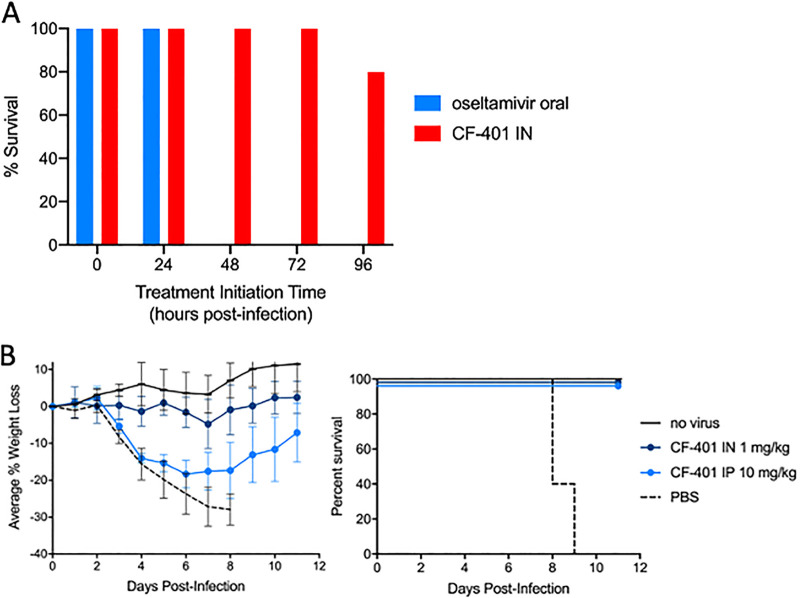
Survival study to assess effective treatment window. (A) Mouse cohorts (*n* = 5) were infected with 2× LD_50_ H1N1 virus (strain A/Puerto Rico/8/34) and dosed with either oral oseltamivir or i.n. CF-401 at various times postinfection. Percent survival was assessed at day 14 postinfection. Oseltamivir treatments consisted of 10 mg/kg oral administrations given twice daily starting at the indicated time postinfection. CF-401 treatments were a single 1 mg/kg i.n. administration at the indicated time postinfection. (B) Mice (*n* = 5) were subjected to a 10× LD_50_ viral challenge with the mouse-adapted A/California/04/2009 H1N1 influenza strain. At 24 h postinfection, bNAb CF-401 was dosed once at either 1 mg/kg i.n. or 10 mg/kg i.p. The percent change in body weight over 11 days is shown with error bars set at 1 SD (left) and the Kaplan-Meier survival plot (right).

It is of interest to assess how long the effective treatment window is for bNAb airway treatments compared to the current standard-of-care anti-influenza therapy, oseltamivir (Tamiflu). A survival study was performed in the standard H1N1 infection model using a 2× LD_50_ viral challenge with treatments initiated at various times postinfection. As shown in Fig. 7, anti-IAV group I bNAb CF-401 (also known as TRL053 [37]), when delivered at 1 mg/kg i.n., conferred 100% survival when treatment was administered up to 72 h postinfection, and 80% survival observed when treated at 96 h postinfection. In contrast, a standard regimen of oseltamivir was completely ineffective in conferring survival when initiated at any time after 24 h postinfection.

To show the effectiveness of bNAb CF-401 against another H1N1 virus in the mouse infection model, mice were infected with pandemic H1N1 influenza strain A/California/04/2009 at 10× LD_50_ and treated with either 1 mg/kg i.n. or 10 mg/kg i.p. CF-401 (Fig. 7B). Both treatments prevented death, with the 1 mg/kg i.n. regimen showing lower average percentage weight loss compared to that obtained with the 10-fold higher i.p. dose (*P < *0.0001).

### Airway-administered bNAbs achieve lower viral counts and cytokine levels in infected lung tissue than 10-fold higher doses of systemically administered bNAbs.

**FIG 8:**
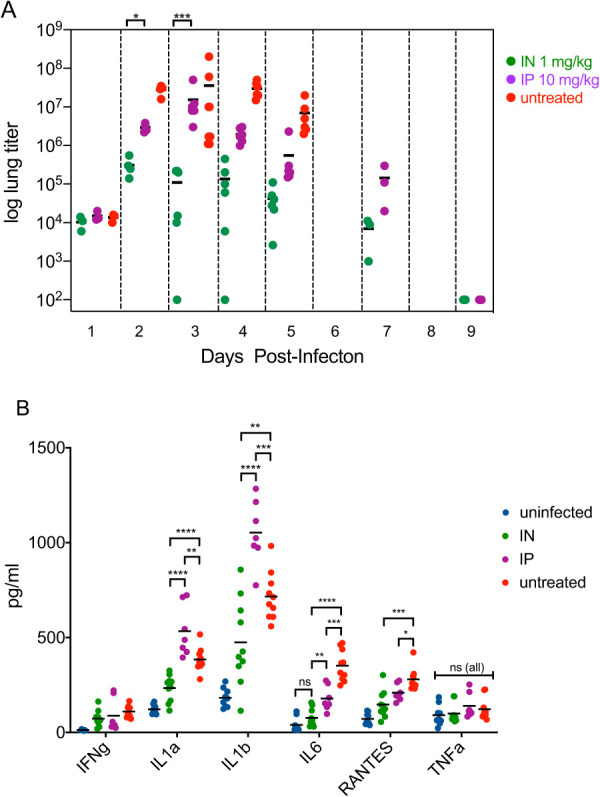
Intranasal delivery reduces inflammatory cytokines and viral titers compared to systemic delivery. (A) Mice cohorts (*n* = 6 for days 3, 4, and 5; *n* = 4 for days 2 and 9, *n* = 3 for days 1 and 7) were infected with 10× LD_50_ H1N1 virus (strain A/Puerto Rico/8/34) and CF-401 was administered at 24 h postinfection by either the i.n. (1 mg/kg) or i.p. (10 mg/kg) routes. For each data point, lungs from a single mouse were harvested and viral titers were determined by plaque assay. The plot shows the viral lung titers over 9 days with the mean value indicated for each cohort of mice. The asterisks indicate the *P* values for comparison of the titers for i.n.-treated versus i.p.-treated mouse cohorts at days 2 and 3. (B) Mice cohorts (*n* = 10 except *n* = 7 for the i.p.-treated group) were infected with 3× LD_50_ H1N1 virus (strain A/Puerto Rico/8/34) and dosed with CF-401 by either the i.n. (1 mg/kg) or i.p. (10 mg/kg) routes at 24 h postinfection. Lungs were harvested 5 days postinfection and cytokine levels were determined from lung homogenates. LOD, limit of detection; ****, *P* < 0.0001; ***, *P* < 0.001; **, *P* < 0.01; *, *P* < 0.05; ns, not significant.

We compared the abilities of both systemic- and airway-delivered bNAb therapies to reduce the viral loads in the lungs of infected mice by directly measuring the amount of plaque-forming units in the lung tissue at various times postinfection. In mice infected with 10× LD_50_ H1N1 virus and treated at 24 h postinfection, i.n. administration of 1 mg/kg CF-401 reduced the viral loads at all times tested compared to a 10-fold higher dose of the same antibody delivered via the systemic route (Fig. 8A).

In mice infected with 3× LD_50_ H1N1 virus and treated at 24 h postinfection, levels of four of the six proinflammatory cytokines tested (IL-1α, IL-1β, IL-6, and RANTES) measured in infected lungs at 5 days postinfection were significantly suppressed in airway-treated mice relative to both those of untreated mice as well as systemically treated mice dosed at 10-fold higher levels for three of them. Notably, the levels of both IL-1α and IL-1β in systemically treated infected mice were significantly increased above levels measured in untreated infected mice. This suggests that systemic administration of anti-influenza bNAbs may result in an increase in symptoms that are associated with high proinflammatory cytokine levels.

### Low levels of antibody needed for effective airway treatment allow combinations of bNAbs to achieve broad anti-influenza coverage.

Due to the enhanced efficacy observed for bNAbs delivered via the airway, relatively low doses are required to treat an influenza infection. This finding creates the possibility of forming effective therapeutic low-dose combinations of antibodies that are each broadly reactive against one of the three prevalent types of influenza that infect humans. We have developed a set of three neutralizing anti-HA-stalk antibodies with specificities for IAV group 1 (TRL053 or CF-401) (37), IAV group 2 (TRL579 or CF-402) (37), and IBV (TRL849 or CF-403) (38), with the equimolar combination of these three antibodies termed CF-404.

**FIG 9:**
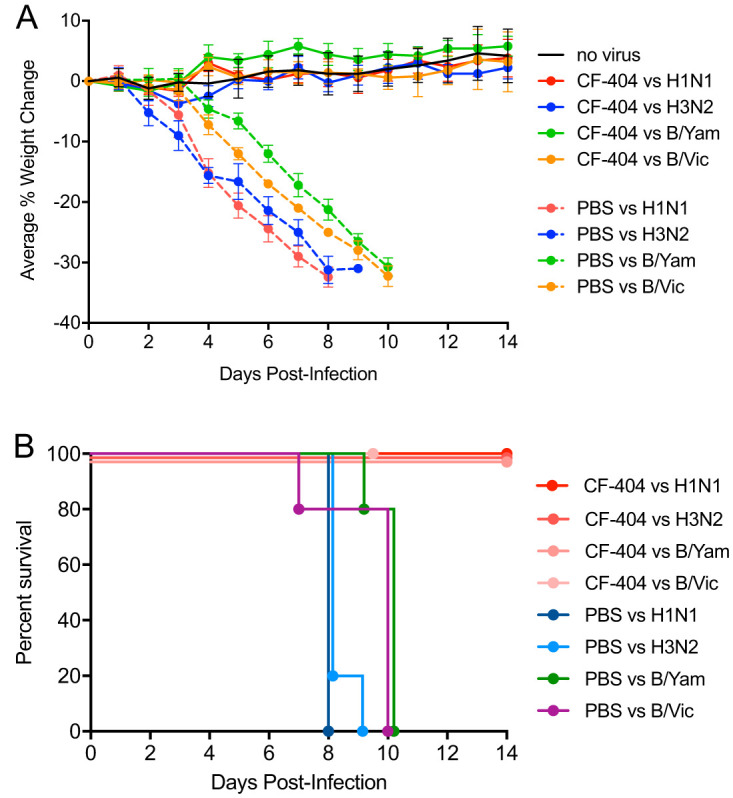
Combination of three bNAbs achieves broad anti-influenza coverage. The triple bNAb combination CF-404 was tested at 3 mg/kg i.n. at 24 h postinfection against dosing cohorts (*n* = 5) infected with 10× LD_50_ viral challenges of four different influenza strains: H1N1 influenza strain A/Puerto Rico/8/1934; H3N2 influenza strain A/Victoria/361/2011; IBV strain B/Florida/04/2006 (Yamagata lineage); IBV strain B/Malaysia/2506/2004 (Victoria lineage). For each virus tested, a control group of PBS-treated mice was included. Panels show the percent changes in body weight over 14 days with error bars set at 1 SD (A) and the Kaplan-Meier survival plot (B).

We tested the ability of the triple bNAb combination CF-404 to treat groups of mice that were separately infected with one of four different influenza strains representing IAV group 1, IAV group 2, and the IBV-Yamagata, and IBV-Victoria strains. As shown in Fig. 9, 3 mg/kg i.n. CF-404 treatment (i.e., 1 mg/kg of each of the three individual bNAbs) dosed at 24 hpi was effective in protecting mice from weight loss and death caused by infections with 10× LD_50_ inoculums of each of the four viruses.

### Airway delivery via the intranasal and nebulization routes yields comparable activities in the mouse influenza infection model.

Delivery of antibodies to the airway can be achieved in several ways. Nebulization is proving to be a feasible method to achieve deposition of biologically active protein therapeutics to the airway in the clinical setting (22). In particular, vibrating mesh nebulizers have distinct advantages for the airway delivery of protein therapeutics (39, 40). To test whether delivery to the airway via nebulization could achieve the same degree of efficacy we observed with i.n. delivery, we performed a comparative efficacy study in the murine influenza infection model in which mice were infected with 3× LD_50_ H1N1 virus and treated at 24 h postinfection. A dose response was measured in groups of infected mice that were dosed with the triple bNAb combination drug CF-404 either via the i.n. route or by inhalation of nebulized material via a vibrating mesh nebulizer. A systemically administered cohort (5 mg/kg i.p.) was included along with two control groups (uninfected and infected but untreated).

**FIG 10:**
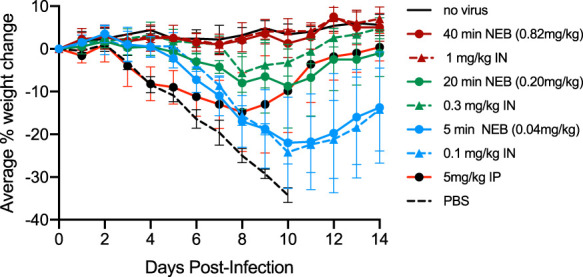
CF-404 administered by nebulization is as effective as intranasal delivery. Mice were infected with 3× LD_50_ H1N1 virus (strain A/Puerto Rico/8/34) and treatment group cohorts (*n* = 5) were dosed at 24 h postinfection with CF-404 at the indicated single doses delivered by the i.n. route, inhalation via nebulization, or by the i.p. route. Also included were included two control groups (no virus and no treatment). The graph shows the average percent changes in body weight over 14 days with error bars set at 1 SD. All mice survived in all treatment cohorts except for the no treatment cohort (PBS), where all mice died between day 8 and day 11 postinfection.

At the highest i.n. and airway-delivered nebulized doses (1 and 0.82 mg/kg, respectively), the treated mouse weight-loss profiles appeared indistinguishable from those of uninfected mice (Fig. 10). The delivered dose of CF-404 (expressed in mg/kg) was determined by HA enzyme-linked immunosorbent assay (ELISA) of mouse lung tissues that were collected and homogenized immediately after aerosol exposure. Results for the groups receiving the lower doses track closely, with comparable weight-loss profiles for i.n. and nebulized groups receiving similar lung-deposited doses. Again, relatively low doses were required for efficacy for the airway-treated mice (both i.n. and nebulization) compared to the systemic-administration control group receiving the 5 mg/kg dose i.p., which yielded a weight-loss profile comparable to that obtained using an approximately 25-fold lower nebulized lung-deposited dose.

## DISCUSSION

**FIG 11:**
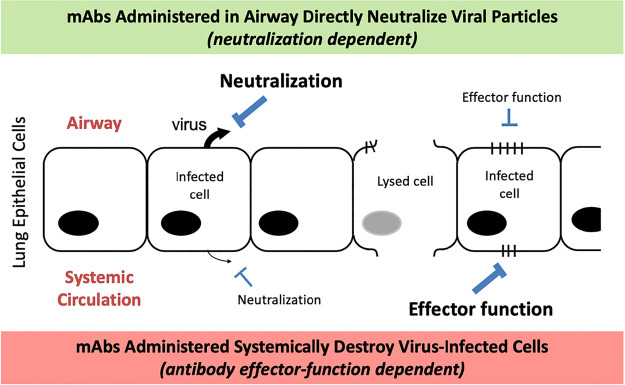
Polarized lung epithelial cell topology dictates differential treatment outcomes for influenza bNAbs administered via different routes. Depiction of lung epithelia with the apical side facing the airway (top side of diagram) and the basolateral side facing the systemic circulation (bottom side of diagram). In the airway-facing side, bNAbs directly neutralize the virus, while on the systemic side bNAbs recognize viral antigens displayed on infected cells and kill the infected cells by recruiting effector cells through Fc gamma receptor engagement.

There are a number of innate and adaptive anti-influenza viral defense mechanisms active in the mucosal layer of the lung epithelia (41). However, when the defenses fail to provide adequate protection, newly replicated influenza virions are almost exclusively shed from the apical (airway-facing) side of infected lung epithelial cells (42) (Fig. 11). The asymmetric distribution of virions at the airway side of the lung epithelia, both at the beginning of the infection and persisting once the infection progresses, provides a rationale for administering anti-influenza HA antibodies to the airway side of the lung epithelia. It also provides a reason for why the neutralization attribute is critical for maximal efficacy of airway-delivered bNAbs, as neutralization of viral infectivity prevents the infection of lung epithelial cells, thereby effectively suppressing the initiation, and therefore the extent, of the viral infection.

Transport of therapeutic proteins across the lung epithelial layer is relatively inefficient in either direction. Only a small fraction of airway-deposited protein therapeutics cross the lung epithelial barrier and enter the bloodstream (43). Similarly, only small amounts of systemically administered protein therapeutics cross the lung epithelial barrier and enter the airway (29). As a result, the vast majority of systemically administered antibodies primarily access the infected lung epithelial cells from the basolateral side. There they recognize viral antigens displayed on infected lung epithelial cells and engage NK and macrophage cells through the effector functions encoded by the antibody’s Fc region (9, 15), thereby facilitating the killing of virally infected cells. This provides an explanation for why effector function is critical for bNAb efficacy when administered through systemic routes. It also provides a rationale for the observation that neutralization is a relatively unimportant attribute for systemically administered bNAbs, as there are comparatively few viral particles at the basolateral side of the lung (42). Because systemically administered bNAb doses are diluted throughout the vascular system of the treated host, relatively high doses must be administered to achieve effective bNAb concentrations at the site of action at the lung basolateral surface. This provides a rationale for the observed large differential in the dose levels required for efficacy between the airway-delivered and systemic-delivered anti-influenza bNAbs.

Consistent with this idea, we have observed a reproducible difference in the kinetic weight-loss profiles of airway-dosed versus systemic-dosed virally infected mice. An example of this weight-loss profile differential can be seen in Fig. 1A, comparing the low (0.1 mg/kg) dose of the airway-delivered bNAb versus the high (10 mg/kg) dose of the systemically administered bNAb. While the AUC analyses of these two curves yield similar values, the overall shapes are clearly different. The airway-administered low-dose profile shows initial weight-loss protection that persists until day 6 postinfection, followed by weight loss until the mice ultimately begin recovering at day 11. In contrast, the systemically dosed profile shows an initial weight loss indistinguishable from that of untreated mice which begins to stabilize at day 4 postinfection and begins recovering at day 7. Similar differential weight-loss patterns were observed in other experiments where systemic and airway administrations were compared (see Fig. 1B, Fig. 3, Fig. 4C, and Fig. 10). The two-log viral titer differential observed at day 3 postinfection (Fig. 8A) demonstrates that infections taking place in systemically treated mice during the early times posttreatment were much more intense compared to the infections in airway-treated mice. These intense infections, with associated elevated cytokine levels, helped drive appetite suppression and the accompanying early weight losses seen in both the untreated and systemically treated mice.

The different weight-loss profiles seen between the two routes of administration and the day-3 viral titer differential may be explained by the fact there is a lag in the ability of the systemically delivered antibodies to exert their therapeutic effect because they primarily work by killing infected cells via effector-function-mediated cytotoxicity (9, 15). Because cells must first become infected in order to engage the systemically administered bNAb’s antiviral mechanism, systemically administered bNAbs exert their therapeutic effect relatively late in the overall infection cycle. In contrast, airway-delivered bNAbs directly neutralize the virus before infection rounds occur, thereby limiting the number of cells that become infected. The relative potency enhancement of airway-delivered bNAbs can be explained in part by attenuation of the amplification of the viral infection by direct neutralization of the virus at an early stage in the viral infection cycle, thereby limiting the rounds of cellular infection. Under experimental dose-limiting conditions where the weight-loss profile can be observed (e.g., the 0.1 mg/kg i.n. dose in Fig. 1A), the protection afforded by the low dose of airway-delivered bNAb is not high enough to completely stop the infection and the virus ultimately breaks through over time, resulting in a delayed maximal infection giving rise to the observed kinetic weight-loss profile. But at higher doses, airway-delivered bNAbs neutralize a sufficient amount of virus present in the airway to protect the host from any weight loss due to the viral challenge.

While the outcomes for mice treated via the airway with the nonneutralizing antibody were not nearly as good as those treated with the neutralizing antibody, some of the nonneutralizing antibody-treated mice did survive, in contrast to the 0% survival rate for the untreated control mice (Fig. 3A and B). This indicates that the neutralizing attribute provides a strong component to airway effectiveness, but that other attributes may be playing a role as well.

The IgG format appears to be more effective than the Fab format in treating H3N2 infection intranasally when tested in the dose-response experiment shown in Fig. 4D. This difference is only significant at the lowest doses tested (0.1 mg/kg) (*P < *0.0001). It is not clear whether this difference is based on the presence of the Fc or the bivalent binding (and associated avidity enhancement) of the full IgG format. However, the somewhat diminished airway activity of the D265A mutant shown in Fig. 4C may be consistent with Fc gamma receptor binding playing a role, because the D265A mutation abolishes binding to all Fc gamma receptors in mice (44). It may be that the nonmutated form of the full-length IgG antibody binds to Fc gamma receptors on cells lining the airway tissue, prolonging the antibody airway residency time enough to confer apparent enhanced activity relative to the mutant D265A IgG form or the Fab forms (which have no Fc region).

The potency advantage of airway over systemic administration for therapeutic anti-influenza antibodies was demonstrated originally with human gamma globulin (17), and later using monoclonal anti-HA-stalk antibodies (28, 29) for IAV infections. In a different respiratory viral model system, the cotton mouse model of RSV infection using IVIG as the therapeutic agent, a similar large efficacy differential was observed with airway treatment outperforming systemically administered treatments by 160-fold (45). Similar to the influenza viral system (43), RSV viral particles also infect and bud from the apical side of the lung epithelial layer during viral infections (46). The significant efficacy advantage afforded by airway delivery of therapeutic antibodies may hold for any respiratory viral system (such as SARS-CoV-2) where the virus exhibits asymmetric replication directed to the apical surface of lung epithelium.

Inflammatory cytokine levels are elevated in influenza-infected patients, causing discomfort, pain, and, in severe cases, life-threatening symptoms (47). As shown in Fig. 8B, systemic delivery of bNAbs to treat influenza infections caused the proinflammatory cytokines IL-1α and IL-1β levels to rise even higher than those seen in untreated infected animals. Because inflammatory cytokine production can be promoted by antibody engagement of Fc gamma receptors in conjunction with other stimulatory signals (48), the systemic administration of effector-function-positive HA-specific bNAbs to mice undergoing a proinflammatory influenza infection may cause heightened proinflammatory cytokine levels. In contrast, the mechanism of action of pulmonary-delivered bNAbs to treat influenza is independent of effector function and results in significantly lower levels of inflammatory cytokines compared to systemic delivery. The ability of airway-delivered bNAbs to suppress the levels of inflammatory cytokines is a positive aspect of this treatment option. This is an important factor when considering that influenza patients who may benefit from this treatment include immunocompromised or elderly individuals whose disease severity closely correlates with cytokine levels (49).

Because the antiviral mechanisms of airway- and systemic-administered bNAbs are so different, we tested whether simultaneous coadministration of the same bNAb via the two different routes would provide a heightened benefit. The data presented in Fig. 5 show that systemic doses at levels well below those needed for activity as a single systemically administered agent can significantly enhance the activity of a coadministered airway-delivered low bNAb dose. It appears that systemic bNAb doses that are too low to effectively treat ongoing high-level infections by themselves can augment therapies that limit the extent of viral infection (such as airway-delivered bNAbs). If the viral infection challenge is lowered enough (either by limiting the inoculum or limiting the infection level through viral neutralization by airway-localized bNAbs), it appears that only relatively low doses of systemically delivered bNAbs are needed to decrease the amplification of the viral replication cycle, presumably through the killing of infected cells. It is possible that prior exposure to influenza (including vaccination) induces sufficient systemic neutralizing antibody to potentiate the airway-localized antibody. If not, then it may be beneficial to treat patients with bNAbs simultaneously via both the airway and systemic routes. Additionally, airway delivery of bNAbs might be productively combined with currently marketed small molecule anti-influenza therapies, such as neuraminidase or polymerase inhibitors (50), which have very different mechanisms of action.

Recent clinical trials testing HA stalk-binding antibody therapies for treating influenza employ systemic administration of a single bNAb (51–54). In *in vivo* IAV challenge trials in humans, efficacy of a systemically administered bNAb requires doses on the order of 40 to 50 mg/kg or ∼3 g for an average-sized 70-kg adult patient (54). As a result, this therapeutic approach is limited in practice to a single bNAb, due to the high costs of goods and the administration volume that can be readily delivered. However, because bNAbs delivered via the airway can effectively prevent and treat influenza infections when delivered at doses on the order of 1 mg/kg in mouse models, it is of interest to consider airway administration of a combination of relatively small amounts of bNAbs in order to achieve potent protection against all influenza viruses that infect humans. We have created a bNAb combination therapeutic termed CF-404, which is comprised of three bNAbs that have reactivities against IAV group 1 (CF-401), IAV group 2 (CF-402), and both lineages (55, 56) of IBVs (CF-403). Each individual bNAb that comprise the CF-404 combination was selected based on its ability to neutralize a broad range of viruses in their respective viral types with high potency (37, 38). Although the dimeric IgA format, which is naturally found in mucosal secretions, could be considered, the IgG format was chosen because recombinant dimeric IgA is not easily produced at scale (57). When administered to mice at 3 mg/kg (1 mg/kg of each constituent bNAb) via the airway, CF-404 is highly effective against high viral challenges (10× LD_50_) of either IAV group 1, IAV group 2, IBV-Victoria lineage, or IBV-Yamagata lineage (Fig. 9). By exploiting the high potency afforded by airway administration, it is possible to create a single therapeutic agent that provides broad coverage against all classes of circulating influenza viruses that infect humans at a feasibly administrable dose.

For successful administration of nebulized protein biologics to the airway, it is important to establish optimal formulation and nebulization conditions that stabilize the protein during the nebulization process so that the integrity of the protein deposited in the lung is maintained (39, 58, 59). We have developed suitable nebulization conditions for the triple bNAb therapeutic combination CF-404 and have used nebulized CF-404 to successfully treat H1N1 infections in the mouse influenza infection model (Fig. 10). The efficacies of intranasally administered and nebulized inhaled treatments were found to be comparable at equal lung-deposited doses. While the majority of the studies presented here were performed using i.n. administration, similar results are likely to be achievable with nebulized bNAbs dosed at comparable levels, since the lung concentration of the MAb is similar from both routes of administration. In one published study, even though an aerosol-delivered bNAb was therapeutically effective, it was found to penetrate less deeply into the mouse airway system than those delivered intranasally (29). However, the specific details of how the aerosol is generated (e.g., droplet size) greatly affect the extent to which aerosols are deposited into the lung tissue compartments (60), so it is difficult to directly compare our results with those of that study due to uncontrolled differences in specific experimental details.

While single bNAbs that neutralize most IAV isolates covering both groups 1 and 2 have been developed and are being tested clinically (51, 52, 54), none of these bNAbs can neutralize IBV strains. Although IBV has not caused pandemics to date, in some years IBV can be the predominant circulating influenza strain in certain geographic regions (61–63) and IBV infections can be as severe as or even more severe than infections due to IAV (64), especially in pediatric populations. For example, in the 2010 to 2011 season, IBV caused 25% of the influenza cases, while it was the cause of 38% of the pediatric deaths (65). Additionally, the well-established anti-influenza neuraminidase inhibitor oseltamivir is less effective in treating IBV infections than IAV infections (66), although this issue is addressed with a class of recently introduced viral polymerase inhibitors (50). Therefore, because the airway delivery route is highly effective and enables the combination approach, we include the anti-IBV bNAb CF-403 (also known as TRL-849 [38]) in the antibody combination CF-404 to address prevalent and sometimes deadly IBV infections. IBV coverage is also a feature of a recently reported multispecific anti-influenza biologic (joined camelid domains) that provides broad coverage across multiple influenza viral types in preclinical studies (67).

By exploiting the high efficacy of airway delivery, which allows for low doses, we have developed the novel triple antibody combination therapeutic, termed CF-404, that is designed to prevent and treat infections caused by IAV (both groups 1 and 2) and IBV (both Victoria and Yamagata lineages). Compared to the relatively narrow strain coverage of a single bNAb delivered using conventional systemic delivery, which requires a high dose, it is projected that exceptionally broad and potent coverage against strains that cause most influenza infections in humans can be achieved using the inhaled bNAb combination therapeutic candidate CF-404.

## MATERIALS AND METHODS

### Antibodies.

All the MAbs used in this study were produced as recombinant MAbs and purified from HEK293 cell culture supernatants by standard methods. The protein sequences for CR6261* and CR8020* match those of the CR6261 and CR8020 MAbs published by Crucell (PBD files 3GBN and 3SDY, respectively). The protein sequences for 5A7* match those of the 5A7 MAb published by Yasugi et al. (32). The three antibodies making up the CF-404 antibody cocktail, i.e., antibodies CF-401 (TRL053 [37]), CF-402 (TRL579 [37]), and CF-403 (TRL849 [38]), were generated using the Trellis CellSpot technology. Antibody 6P15 is a phage-display-derived antibody that specifically binds H3N2 influenza strains (A/Aichi/2/1968, A/Hong Kong/1/1968, A/Victoria/3/1975, A/Beijing/47/1992, A/Johannesburg/33/1994, A/Sydney/5/1997, A/Panama/2007/1999, A/Wyoming/3/2003, A/Wisconsin/67/2005, A/Perth/16/2009, and A/Brisbane/10/2007) but does not neutralize the four H3N2 strains tested (A/Hong Kong/1/1968, A/Sydney/5/1997, A/Wisconsin/67/2005, and A/Victoria/361/2011). Antibody 6P15 does not bind the tested H1N1 strains (A/Puerto Rico/8/1934, A/Brisbane/59/2007, and A/California/04/2009), H7N7 strains (A/England/268/1996 and A/Netherlands/219/2003), or IBV strain (B/Florida/4/2006).

### Virus neutralization assay.

Antibodies and viruses (100× 50% tissue culture infective dose [TCID_50_]/well) were preincubated for 1 h at room temperature prior to inoculation of 96-well plates of confluent MDCK cells. The plates were incubated for 1 h at 37°C under 5% CO_2_ to allow for adsorption. Plates were washed twice with PBS and overlaid with 1× minimal essential medium (MEM) containing l-1-tosylamido-2-phenylethyl chloromethyl ketone (TPCK)-trypsin. After approximately 20 to 24 h, the cells were fixed with 80% acetone and then blocked with 5% nonfat milk, followed by 3% hydrogen peroxide. The cells were incubated with a 1:1,500 dilution of biotin-conjugated mouse anti-NP (Millipore), followed by a 1:5,000 dilution of secondary horseradish peroxidase (HRP)-conjugated streptavidin (Millipore). Peroxidase substrate (Sigmafast OPD, Sigma-Aldrich) was added to the wells, reactions were stopped with 3 M HCl, and the absorbance at 492 nm was read on a plate reader.

### Hemagglutination inhibition assay.

The hemagglutination inhibition (HI) assay was performed as described previously (68).

### Viruses.

The following viruses were acquired from BEI resources: A/California/07/2009 (BEI NR-13663) and A/Victoria/361/2011 (BEI NR-44022). We thank Peter Palese for A/Puerto Rico/8/1934, A/Hong Kong/1/1968, A/Hong Kong/1/1968 (2:6) (X31), B/Malaysia/2506/2004 (Vic), B/Florida/04/2006 (Yam), B/Victoria/2/1987, and B/Yamagata/16/1988. Viruses were propagated in specific pathogen-free 10-day-old embryonated chicken eggs from Charles River, mouse adapted (except for PR8), and the mouse LD_50_ was determined according to reference 69. In line with reports (70), mouse LD_50s_ in PFU were as follows: 1.2 × 10^1^ for A/Puerto Rico/8/34, 2.5 × 10^2^ for A/California/07/2009, 6.6 × 10^2^ for A/Victoria/361/2011, 4.2 × 10^4^ for X-31 (A/Hong Kong/68 [2:6]), 1.5 × 10^3^ for B/Florida/04/2006, and 4.2 × 10^3^ for B/Malaysia/2506/2004.

### Mouse influenza infection model.

Female BALB/c mice were ordered from the Jackson Laboratory and were 6 to 8 weeks old at the time of the experiments. Mice (*n* = 5 per group) were anesthetized with a ketamine/xylazine mixture and subsequently infected intranasally using 50 μl of PBS containing various amounts of the 50% lethal dose (LD_50_) of various influenza viruses. The anesthetized mice were treated at various times postinfection with various amounts of bNAbs in PBS delivered either as a 0.1-ml bolus by i.p. injection or as a 0.05 ml i.n. instillation. The mice were monitored over 14 days for morbidity, weight loss, and date of death. The typical indications of pain and distress with influenza infection in mice are ruffled hair (piloerection), loss of appetite, weight loss, and hind limb paralysis. Since one indication is not sufficient to determine a lethal outcome of infection, we utilized a scoring system to justify early removal. Details of the scoring system that were utilized can be found in reference 71. All animal procedures performed in this study are in accordance with Institutional Animal Care and Use Committee (IACUC) guidelines and have been approved by the IACUC of ContraFect Corporation.

### Lung viral titer assay.

Infected mice were euthanized and the whole lungs were removed, resuspended in 2 ml of 1× PBS containing protease inhibitors (Complete Ultra tablets, Roche), and homogenized using a Precellys 24 homogenizer (Bertin Technologies). Samples were centrifuged to remove tissue debris and supernatants were transferred to a new tube. Viral titers were determined by plaque assay in MDCK cells followed by immunostaining. For the plaque assays, MDCK cells were seeded on 6-well plates 12 to 18 h prior to the assay. The virus suspensions were serially diluted in 1× PBS and 1 ml of the virus dilution was used to infect each well of MDCK cells for 1 h at 37°C. After the incubation, the virus was removed, the cells washed twice with 1× PBS and 3 ml of Avicell mix (1.2% Avicell in 1× MEM with bovine serum albumin [BSA] and trypsin) was added to each well. Plates were incubated at 37°C for 48 h (influenza A viruses) or at 34°C for 72 h (influenza B virus). After incubation, cells were fixed with 3 ml of 4% paraformaldehyde (PFA). For the immunostaining, the cells were blocked for 20 min with 5% milk in 1× PBS and the primary (test antibodies) and secondary antibody (anti-human IgG-HRP) were added sequentially. After the final wash, 500 μl of TrueBlue was added to visualize and count the plaques.

### Inhalation delivery.

Mice were placed into individual nose-only restraint tubes and connected to a radial nose-only inhalation exposure chamber (In-Tox Products, Moriarity, NM, USA) for aerosol exposure. CF-404 was aerosolized with a vibrating mesh nebulizer (AeroNeb-Solo, Aerogen Ltd., Deerfield, IL, USA). The aerosols were transitioned into a rodent nose-only inhalation exposure chamber. Mice were exposed to aerosolized CF-404 for either 5, 10, 20, or 40 min. Targeted lung-deposited doses were controlled by adjusting CF-404 concentration prior to nebulization, with CF-404 aerosol concentration measured by sampling directly from the breathing zone of the rodent exposure system, and aerosol flow rate. Exposure duration for each dose level was determined by using the predicted targeted lung-deposited dose per mg of body weight. CF-404 aerosol concentrations were determined by HA ELISA. Particle size was determined by using a cascade impactor (In-Tox Products, Moriarity, NM, USA). The impactor substrates were immediately analyzed via differential weight to determine the total particle size distribution and calculated using SigmaPlot software (Systat Software, Inc., San Jose, CA, USA). Using this system, aerosols were produced with a 2.17 mass median aerodynamic diameter (MMAD) and a 2.24 geometric standard deviation (GSD). The delivered dose of CF-404 as expressed in mg/kg was determined by HA ELISA of mouse lung tissues that were collected and homogenized immediately after aerosol exposure.

### Statistical analysis.

All statistical analyses and plot generations were made using Prism 8 software (GraphPad Software, Inc., La Jolla, CA, USA). Area under the curve (AUC) analysis was used to assess the statistical relationships between the percent weight loss profiles resulting from the different treatments. The statistical relationship between two curves was assessed by calculating the mean AUC value, the standard error, and the number of degrees of freedom for each pair and performing the unpaired *t* test with Welch’s correction. The log-rank (Mantel-Cox) test was used to assess the significance of Kaplan-Meier survival curve comparisons. Unpaired *t* test analyses were used to assess the statistical relationships between the cytokine levels (Fig. 8B).
